# Novel Systemic Associations of Idiopathic Epiretinal Membrane Identified via Machine Learning

**DOI:** 10.1016/j.xops.2026.101124

**Published:** 2026-02-18

**Authors:** Ethan Wu, Jessica Jiang, Nasiq Hasan, Katherine Du, Michelle Zhang, Joanna Yao, Kiran Kumar Vupparaboina, Sandeep Chandra Bollepalli, José-Alain Sahel, Jay Chhablani

**Affiliations:** 1 Department of Ophthalmology, UPMC (University of Pittsburgh Medical Center), Pittsburgh, Pennsylvania; 2 Medical Scientist Training Program, University of Pittsburgh School of Medicine, Pittsburgh, Pennsylvania

**Keywords:** All of Us Dataset, Epiretinal membrane, Machine Learning, Systemic comorbidities

## Abstract

**Purpose:**

To discover novel systemic associations that may lead to idiopathic epiretinal membrane (iERM) using interpretable machine learning models.

**Design:**

Large data retrospective case-control study.

**Subjects:**

All of Us Dataset, including a total of 10 380 patients: 2015 iERM patients with 2015 1:1 matched controls, 3175 secondary epiretinal membrane (sERM) patients with 3175 1:1 matched controls.

**Methods:**

Electronic health records of epiretinal membrane (ERM) patients from the All of Us Research Program, a nationwide longitudinal cohort of US adults (data from 6/2016 to 2/2025) were collected. Unsupervised clustering using principal component analysis was performed on the data set to identify distinct patient subgroups. Supervised machine learning models, including gradient-boosted decision trees and logistic regression, were trained to predict iERM.

**Main Outcome Measures:**

Model performance was evaluated using the area under the receiver operating characteristic curve (AUC), while feature importance was assessed using the Gini index for tree-based models and coefficient magnitudes for logistic regression. Additionally, odds ratios for comorbidities associated with both iERM and sERM were estimated using 2 × 2 contingency tables.

**Results:**

Unsupervised clustering of iERM patients revealed 4 distinct subgroups characterized by unique systemic comorbidity profiles, including cardiometabolic, dermatologic, and joint disorder pathways. Clusters demonstrated significant associations with systemic conditions such as hypertension, hyperlipidemia, type 2 diabetes, inflammatory skin conditions, osteoarthritis, and anemia. Supervised models, including logistic regression and gradient-boosted decision trees, achieved AUC values exceeding 0.679 on a testing set. Key predictors of iERM included knee osteoarthritis, hyperlipidemia, essential hypertension, and sensorineural hearing loss, each demonstrating high coefficient magnitudes, Gini importance, and statistically significant odds ratios.

**Conclusions:**

This study challenges conventional distinctions between iERM and sERM, proposing systemic comorbidities as associations to ERM development. The observed associations with cardiometabolic dysfunction, chronic inflammation, and joint and dermatologic disorders suggest that systemic mechanisms may significantly influence ERM pathogenesis. Future studies are necessary to establish causality and explore targeted therapeutic approaches, potentially incorporating anti-inflammatory treatments or cardiovascular risk management to prevent ERM formation. These findings highlight opportunities for personalized risk assessment and preventative interventions based on systemic comorbidity profiles.

**Financial Disclosure(s):**

Proprietary or commercial disclosure may be found in the Footnotes and Disclosures at the end of this article.

Epiretinal membrane (ERM) is a fibrocellular, avascular proliferation that develops along the inner retinal surface, primarily in the macular region.^1^ It is characterized by the preretinal proliferation of myofibroblastic cells and the accumulation of extracellular matrix. While often asymptomatic, ERM can cause retinal distortion and traction, resulting in varying presentations of visual impairment, such as metamorphopsia, reduced visual acuity, and diminished contrast sensitivity.^2^ Epiretinal membrane is commonly diagnosed clinically by retinal OCT imaging, which reveals hyperreflective bands on the retinal surface indicative of membrane formation.^3^

The prevalence of ERM varies across different populations, ranging from 7% in the Blue Mountain Eye Study to 28.9% in the MESA Study.^4^, ^5^, ^6^ The Beaver Dam Eye Study initially reported an 11.8% prevalence of ERM in 1994; however, a 20-year follow-up revealed a higher prevalence of 34.1%.^7^^,^^8^ Ocular conditions, such as diabetic retinopathy, uveitis, retinal detachment, and ocular trauma can lead to secondary epiretinal membrane (sERM).^9^ Epiretinal membrane can also develop idiopathically, though its exact pathogenesis remains unclear. However, it is strongly associated with posterior vitreous detachment, an age-related process affecting the vitreoretinal interface.^10^

Diagnosis of idiopathic epiretinal membrane (iERM) relies on exclusion, requiring careful evaluation to rule out secondary causes. Given its increasing prevalence with age and its strong association with posterior vitreous detachment, understanding the risk factors and systemic associations of iERM is critical for early detection, patient management, and potential therapeutic strategies. In this study, we utilize machine learning models on the publicly available large-scale All of Us data set to reveal previously unrecognized associations and risk factors for iERM. These findings could accelerate early detection efforts and enhance our understanding of underlying mechanisms of iERM development.

## Methods

### Study Design and Participants

This retrospective study utilizes the All of Us data set, a nationwide initiative led by the National Institutes of Health. The objectives, recruitment strategy, study sites, and scientific framework of the All of Us Research Program have been previously detailed.^11^ The program aims to reflect the diversity of the United States by enrolling participants historically underrepresented in biomedical research, including racial and ethnic minorities, rural residents, sexual and gender minorities, and those with limited health care access.^12^ All participants in the All of Us Research Program provided informed consent for the use of their data in research. This study was conducted in accordance with the All of Us Data and Statistics Dissemination Policy and complied with the ethical standards set by the All of Us Research Program Data Use Agreement.

Data extraction was performed through the All of Us Researcher Workbench. We defined our case cohorts as patients aged 18 years or older with a diagnosis of epiretinal membrane. Cases were identified using a combination of keyword searches for disease terms, such as epiretinal membrane or macular pucker, and relevant diagnostic codes from both International Classification of Diseases, Ninth and 10th Revisions.

This study included patients diagnosed with iERM and sERM. Ocular comorbidities were reviewed for each individual, and conditions known to contribute to sERM were identified by name using the All of Us Cohort Builder and categorized as detailed in Table S1 (available at www.ophthalmologyscience.org).^13^, ^14^, ^15^, ^16^ The iERM group comprised 2015 patients with epiretinal membrane and no identifiable secondary cause. The sERM group included 3175 patients with epiretinal membrane attributed to a defined secondary etiology.

Specifically, all participants with an epiretinal membrane diagnosis were first identified, and the date of first recorded ERM diagnosis was designated as the index date. To ensure appropriate temporality, only diagnoses documented prior to the index ERM date were considered qualifying secondary etiologies (Table S1). Because this classification relied on International Classification of Diseases-coded diagnoses, the designation of “idiopathic” reflects the absence of documented secondary ocular conditions in the electronic health record (EHR) rather than definitive exclusion of all possible secondary causes.

To enable appropriate comparisons, we created 2 separate 1:1 age-matched, sex-matched, and race-matched control groups, one for iERM and one for sERM. For each iERM case, all ERM-free participants of the same age, race, and sex were identified, and one was randomly selected as a matched control. This process yielded 2015 controls for the iERM cohort and 3175 controls for the sERM cohort.

Note: Each matched control group was 2 participants smaller than its corresponding epiretinal membrane cohort due to the absence of non-ERM individuals with matching age, sex, and race for 2 cases in both the iERM and sERM cohorts.

### Ethics Statement

This study was a secondary analysis of deidentified data from National Institutes of Health All of Us Research Program. The research was conducted in accordance with the principles of the Declaration of Helsinki. Institutional review board and ethics committee oversight for the All of Us Research Program is provided centrally by the NIH All of Us Research Program Institutional Review Board. Because investigators accessed only deidentified data, informed consent for the specific analyses in this manuscript was not required. All of Us participants provide informed consent for data collection and use within the program.

### Coding and Analysis

Python 3.0, along with relevant libraries such as scikit-learn and pandas, was used for data manipulation and statistical analysis.

### Unsupervised Clustering Approach

Within the cohort of iERM patients, we applied principal component analysis to reduce the dimensionality of comorbidity data diagnosed prior to ERM diagnosis, thus allowing the exploration of potential risk stratification patterns and associations.^17^ Input features included a comprehensive set of comorbid conditions identified from structured diagnostic codes in the EHR. These included, but were not limited to, hypertension, diabetes, autoimmune diseases, degenerative conditions, and other chronic or acute diagnoses. K-means clustering (n_clusters = 4) was performed using the first 50 principal components, which together explained 31.42% of the cumulative variance. The first 2 principal components (PC1 = 4.61%, PC2 = 1.49%) were used solely for visualization of cluster structure.

To select the number of clusters, we evaluated k values from 2 to 8 using silhouette analysis and within-cluster sum of squares (inertia). Although k = 2 produced the highest silhouette score, k = 4 provided reasonable separation with improved clinical interpretability and diminishing improvements in inertia beyond this point (Table S2, available at www.ophthalmologyscience.org); therefore, k = 4 was selected for downstream analyses.

### Supervised Machine Learning Models to Differentiate Comorbidity Data

To distinguish iERM patients from age-matched and sex-matched healthy controls, we implemented 2 supervised machine learning models: logistic regression and gradient-boosted decision trees (GBDTs).

Cohort was randomly partitioned into training (75%) and test (25%) sets using a standard holdout approach. Both models were trained using the full comorbidity feature set and only on the training set. Model performance in identifying iERM was evaluated using area under the receiver operating characteristic curve (AUC) for logistic regression and GBDT. Parameters were not hypertuned to prevent potential overfitting. Feature importance was assessed via coefficient magnitude in logistic regression and Gini importance in GBDT. Model-driven insights were validated by odds ratio analysis, assessing associations between key features and patient clusters. A *P* value threshold of <0.05 was used to determine statistical significance.

Logistic regression was used as a transparent baseline model to estimate linear associations between individual comorbidities and iERM status. In contrast, GBDT was employed to capture nonlinear relationships and higher-order interactions among comorbid conditions, which are common in complex, multimorbid clinical data sets and may not be adequately modeled using linear approaches alone.

### Risk Estimation

To validate the comorbidities associated with idiopathic and secondary ERM, we calculated odds ratios using 2 × 2 contingency tables to quantify to quantify the strength of the association between each comorbidity comorbidities and ERM status. Exact odds ratios and corresponding *P* values were obtained using Fisher exact test.

## Results

### Patient Characteristics

This study analyzed a total of 5190 patients diagnosed with ERM, comprising 2015 patients with iERM and 3175 with sERM. Age-matched and sex-matched control cohorts were established for each patient group. Specifically, 2015 healthy controls without ERM were matched 1:1 with the iERM group (mean age: 71 years), while 3175 controls were similarly matched with the sERM cohort. The majority of patients in both ERM subgroups were White, 86% in the iERM group and 80% in the sERM group. The gender distribution was relatively balanced, with males comprising 52.8% of the iERM group and 50.8% of the sERM group (Table 1).

**Table 1:** Demographic Information of Idiopathic ERM and Nonidiopathic, or Secondary, ERM, and Their Respective Healthy Control Groups

Demographics	Idiopathic (N = 2000–2020)	Idiopathic Control (N = 2000–2020)	Nonidiopathic (N = 3160–3180)	Nonidiopathic Control (N = 3160–3180)
Gender				
Female	1060–1080	1060–1080	1600–1620	1600–1620
Male	940–960	940–960	1560–1580	1560–1580
Race				
American Indian	0–20	0–20	0–20	0–20
Asian	60–80	60–80	100–120	100–120
Black or African American	140–160	140–160	360–380	360–380
I prefer not to answer	0–20	0–20	0–20	0–20
Middle Eastern or North African	0–20	0–20	20–40	20–40
More than one population	40	40	100–120	100–120
Native Hawaiian or Other Pacific Islander	0–20	0–20	0–20	0–20
None indicated	0–20	0–20	0–20	0–20
None of these	0–20	0–20	0–20	0–20
Skip	0–20	0–20	0–20	0–20
White	1720–1740	1720–1740	2540–2560	2540–2560
Age (mean, [SD])	71.0 (8.8)	71.0 (8.8)	70.0 (10.0)	70.0 (10.0)

ERM = epiretinal membrane; SD = standard deviation.

### Unsupervised Learning Reveals Distinct Systemic Comorbidity Clusters in Idiopathic ERM

**Figure 1:**
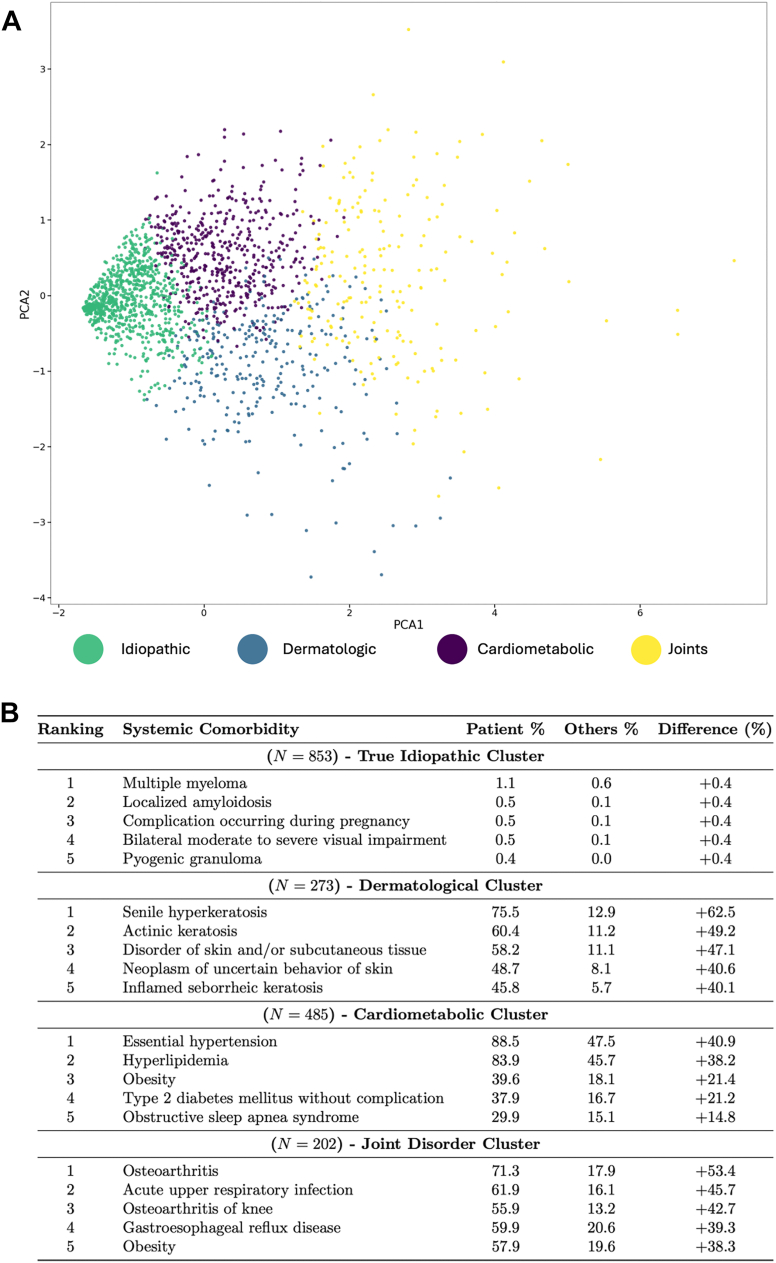
Comorbidity-based PCA-clustering reveals distinct systemic disease idiopathic ERM patient cluster. **A,** Principal component analysis visualization shows patient clusters based on comorbidity profiles. Green = true idiopathic, blue = dermatological cluster, purple = vascular, yellow = joint disorder cluster. **B,** Distribution of unique systemic comorbidities across each cluster. ERM = epiretinal membrane; PCA = principal component analysis.

To investigate systemic disease patterns of 2015 patients with iERM, we applied unsupervised clustering using principal component analysis on all comorbidities diagnosed prior to ERM diagnosis. The unsupervised analysis revealed 4 distinct clusters, each characterized by unique systemic health profiles (Fig 1).

1. The true idiopathic cluster (47.0%) comprised nearly half of the iERM cohort and was characterized by the absence of statistically significant systemic comorbidities compared to other ERM clusters. No single comorbidity was more prevalent in this cluster than in others, suggesting the presence of a subset of idiopathic ERM patients lacking identifiable systemic disease associations.
2. The dermatological cluster (15.1%) exhibited a high prevalence of skin-related conditions, including senile hyperkeratosis (75.5%), actinic keratosis (60.4%) inflammatory dermatosis (44.3%), and benign neoplasm of uncertain behavior of skin (48.7%). This cluster suggests a potential link between dermatologic inflammatory or proliferative disorders and iERM pathogenesis.
3. The cardiometabolic cluster (26.8%) was predominantly associated with cardiometabolic disorders. A substantial proportion of these patients had essential hypertension (88.5%), hyperlipidemia (83.9%), obesity (39.6%), and type 2 diabetes mellitus (37.9%), suggesting a strong metabolic and cardiovascular component.
4. The joint disorder cluster (11.1%) was primarily characterized by musculoskeletal and inflammatory features, including osteoarthritis (71.3%), knee osteoarthritis (61.9%), upper respiratory infection (61.9%), and gastroesophageal reflux disease (59.9%), indicating a potential association between joint degeneration, systemic inflammation, and idiopathic ERM.

While a subset of iERM patients exhibited no significant comorbidities, other clusters demonstrated strong associations with cardiometabolic, dermatologic, and joint-related conditions. These findings highlight the clinical heterogeneity of iERM and suggest multiple, potentially novel pathogenic pathways underlying its development.

### Supervised Learning Differentiates between Idiopathic ERM and Healthy Patients, Pinpointing Specific Systemic Causes

**Figure 2:**
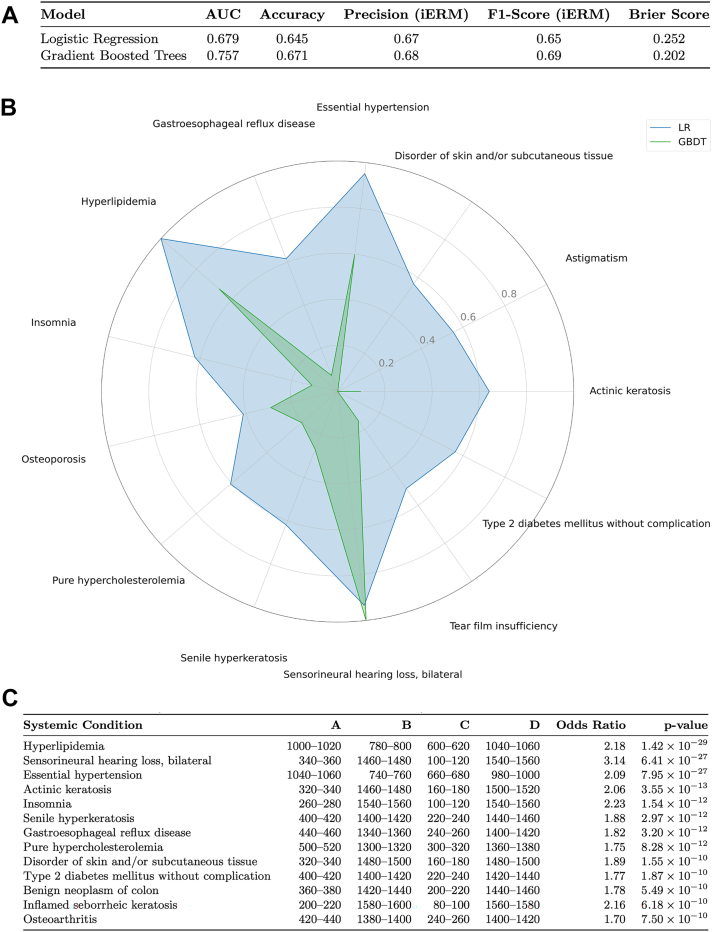
Supervised learning reveals major differences between iERM and matched healthy controls. **A,** Accuracy of logistic regression and GBDT. **B,** Important input features of predictive models to predict iERM. **C,** Odds ratios of top features to differentiate iERM and matched healthy controls. Note: A = iERM cases with condition; B = iERM cases without condition; C = healthy controls with condition; D = healthy controls without condition. AUC = area under the receiver operating characteristic curve; GBDT = gradient-boosted decision tree; iERM = idiopathic epiretinal membrane; LR = logistic regression.

Logistic regression achieved a test set AUC of 0.679, while GBDTs attained an AUC of 0.757, indicating that systemic comorbidities can moderately predict the presence of iERM (Fig 2A).

Analysis of feature importance from both models identified knee osteoarthritis, hyperlipidemia, essential hypertension, and sensorineural hearing loss as the strongest predictors of iERM (Fig 2B). Odds ratio analysis further confirmed the significance of these predictors. Hyperlipidemia was associated with an odds ratio of 2.18 (*P* = 1.42 × 10^–29^), while essential hypertension had an odds ratio of 2.09 (*P* = 7.95 × 10^–27^). Sensorineural hearing loss demonstrated an odds ratio of 3.14 (*P* = 6.41 × 10^–27^), supporting its relevance in iERM risk (Fig 2C).

Dermatologic conditions also revealed significant associations with iERM. Senile hyperkeratosis and actinic keratosis had odds ratios of 1.88 and 2.06, respectively. These findings further suggest that the iERM clusters found through principal component analysis may be linked to systemic metabolic, cardiovascular, musculoskeletal, and dermatologic factors, thus reinforcing the hypothesis that multiple systemic pathways contribute to its development.

### Secondary ERM Patients Share Similar Systemic Causes

Analysis of sERM patients revealed that metabolic, cardiovascular, and inflammatory comorbidities are also highly prevalent in this group. Hypertension, hyperlipidemia, and senile hyperkeratosis had similarly significant odds ratios in sERM patients compared to controls (Table 2).

**Table 2:** Odds Ratios of Secondary ERM Patients vs. Healthy Controls

Condition	A	B	C	D	Odds Ratio	*P* Value
Vitreous degeneration	1320–1340	1840–1860	120–140	2560–2580	13.94	2.45 × 10^–265^
Nuclear senile cataract	1560–1580	1600–1620	260–280	2420–2440	8.78	1.20 × 10^–249^
Cataract	1220–1240	1940–1960	200–220	2480–2500	7.46	2.45 × 10^–181^
Presbyopia	1180–1200	1960–1980	220–240	2460–2480	6.25	5.87 × 10^–157^
Tear film insufficiency	980–1000	2180–2200	160–180	2520–2540	6.93	5.56 × 10^–141^
Vitreous opacities	740–760	2420–2440	60–80	2600–2620	10.25	3.44 × 10^–131^
Myopia	900–920	2260–2280	140–160	2540–2560	6.66	8.66 × 10^–127^
Astigmatism	760–780	2400–2420	120–140	2560–2580	5.97	1.38 × 10^–100^
Hyperlipidemia	1960–1980	1200–1220	940–960	1740–1760	3.0	4.89 × 10^–94^
Retinal disorder	440–460	2720–2740	20–40	2660–2680	15.91	5.76 × 10^–92^
Senile cataract	660–680	2500–2520	100–120	2580–2600	6.19	6.49 × 10^–90^
Visual disturbance	660–680	2500–2520	140–160	2540–2560	4.8	7.12 × 10^–74^
Ocular hypertension	540–560	2620–2640	80–100	2600–2620	6.14	1.08 × 10^–73^
Blepharitis	560–580	2600–2620	100–120	2580–2600	5.6	3.94 × 10^–72^
Essential hypertension	1940–1960	1220–1240	1060–1080	1600–1620	2.39	1.59 × 10^–60^
Secondary cataract	340–360	2820–2840	20–40	2660–2680	9.77	1.98 × 10^–60^
Vitreous hemorrhage	260–280	2900–2920	0–20	2680–2700	24.28	1.35 × 10^–58^
Disorder of refraction and/or accommodation	520–540	2640–2660	100–120	2580–2600	4.56	2.63 × 10^–57^
Cystoid macular retinal degeneration	240–260	2920–2940	0–20	2680–2700	25.09	3.84 × 10^–55^
Inflammatory dermatosis	860–880	2300–2320	300–320	2380–2400	2.94	2.14 × 10^–54^

A = secondary ERM cases with condition; B = secondary ERM cases without condition; C = healthy controls with condition; D = healthy controls without condition; ERM = epiretinal membrane.

The prevalence of vascular, metabolic, and inflammatory comorbidities was nearly identical between idiopathic and secondary ERM patients. Hypertension, hyperlipidemia, osteoarthritis, acute keratosis, and sensorineural hearing loss were present in both iERM and sERM groups, at varying notable proportions (Table 3).

**Table 3:** Similarities and Differences between Common Systemic Conditions of Idiopathic ERM and Secondary ERM

Condition	Idiopathic ERM	Secondary ERM
Essential hypertension	56.8%	57.9%
Hyperlipidemia	52.9%	59.3%
Pure hypercholesterolemia	29.8%	35.3%
Gastroesophageal reflux disease	25.1%	31.4%
Obesity	23.0%	29.6%
Benign neoplasm of colon	22.8%	26.7%
Senile hyperkeratosis	22.2%	27.9%
Osteoarthritis	21.1%	24.8%
Type 2 diabetes mellitus without complication	21.0%	29.2%
Gastroesophageal reflux disease without esophageal lesion	20.1%	20.6%
Acute upper respiratory infection	19.7%	27.8%
Inflammatory dermatosis	19.6%	26.5%
Actinic keratosis	19.5%	20.3%
Disorder of skin and/or subcutaneous tissue	18.0%	21.5%
Nuclear senile cataract	17.7%	45.4%
Allergic rhinitis	17.3%	22.5%
Sensorineural hearing loss, bilateral	17.1%	17.4%

ERM = epiretinal membrane.

## Discussion

This study identifies significant associations between ERM and systemic comorbidities in both iERM and sERM, generating the hypothesis that broader systemic disease mechanisms may be linked to ERM risk. The identification of distinct patient clusters within iERM highlights multiple potential pathways associated to iERM development, including cardiometabolic, inflammatory, and dermatologic processes. At the same time, the strong overlap in comorbidities between iERM and sERM suggests that systemic comorbidities are common in both iERM and sERM and may influence susceptibility or fibrotic remodeling even when a local ocular trigger is present.

Unsupervised clustering of iERM patients identified 4 distinct groups with unique systemic comorbidity profiles, suggesting that iERM may arise from multiple biological pathways. A distinct cluster comprising nearly half of iERM patients exhibited no significant comorbidities, reinforcing the possibility that a subset of iERM cases may develop in the absence of systemic contributions. However, the remaining clusters demonstrated associations with cardiometabolic, metabolic, musculoskeletal, inflammatory, and dermatologic conditions. The cardiometabolic cluster was associated with systemic conditions of microvascular dysfunction, such as essential hypertension, hyperlipidemia, obesity, and type 2 diabetes. Chronic hypertension and hyperlipidemia may disrupt the integrity of the blood–retina barrier, leading to inflammatory changes and increased migration of Müller cells, a key process in ERM formation.^18^^,^^19^

Clustering also identified an unexpected yet significant dermatologic component in iERM, with a subgroup of patients exhibiting a high prevalence of hyperkeratosis, inflammatory dermatoses, benign neoplasms of the skin, and actinic keratosis. Although the physiological connection between dermatological conditions and epiretinal membrane remains unclear, signaling from chronic inflammation may be mutual. Many dermatologic conditions identified in this cluster involve prolonged immune activation, particularly involving cytokines such as interleukin-6 and tumor necrosis factor-alpha, which have been implicated in retinal inflammation and fibrosis.^20^^,^^21^ This raises the possibility that systemic inflammatory pathways, rather than local retinal pathology alone, may contribute to ERM formation in certain patient populations. Many of the dermatologic conditions identified are linked to ultraviolet (UV) radiation exposure, and UV radiation-induced oxidative stress has also been associated with other ocular conditions such as cataract, glaucoma, and age-related macular degeneration, further supporting that systemic inflammatory pathways may contribute to ERM formation.^22^^,^^23^ The presence of dermatologic conditions and epiretinal membrane formation in a subset of patients raises the hypothesis that UV-exposure-linked conditions are associated with ERM risk.

The joint disorder was dominated by musculoskeletal and hematological conditions, most notably osteoarthritis and anemia, supporting the view that systemic processes contribute to iERM. Once regarded as a purely mechanical, joint-limited disorder, osteoarthritis is now recognized as a disease driven by low-grade systemic inflammation and dysregulated by innate and adaptive immune responses.^24^ Chronic anemia, in contrast, can reduce retinal oxygen delivery, triggering hypoxia-induced oxidative stress and extracellular matrix remodeling, which has been associated with a higher prevalence of ERM in other studies.^25^^,^^26^ Together, these links implicate micro-inflammatory and hypoxic-stress pathways as plausible mechanisms connecting the joint cluster morbidities to iERM formation.

Since clustering was performed on EHR-coded comorbidities, cluster membership across the cohort may be influenced by noncausal factors such as age, overall comorbidity burden, health care utilization, and socioeconomic factors affecting access to care and diagnosis capture. These considerations are particularly relevant for the dermatologic and joint disorder clusters, as several driving diagnoses like actinic keratosis, benign skin neoplasms, and osteoarthritis are strongly age-associated and may be sensitive to screening intensity, encounter frequency, and coding practices. In addition, socioeconomic and occupational factors may influence both UV exposure and the likelihood of receiving dermatologic diagnoses.

Supervised learning models reinforced the significance of these systemic conditions in iERM. Logistic regression and gradient-boosted decision trees demonstrated moderate predictive accuracy. Both achieved AUC values above 67.9%, indicating that comorbidity profiles may be partially used to identify patients at risk for iERM. Feature importance analysis highlighted knee osteoarthritis, hyperlipidemia, essential hypertension, and sensorineural hearing loss as key predictors. The association with cardiovascular risk factors further underscores the role of systemic cardiometabolic dysfunction in ERM development.

Beyond inflammatory and systemic cardiometabolic factors, the link between iERM and sensorineural hearing loss suggests shared neurodegenerative pathways between the retina and the cochlea. Both organs are highly metabolically active, depend on dense capillary networks, the superficial and deep retinal plexuses in the macula and the stria vascularis within the cochlea and are protected by blood–tissue barriers with limited collateral flow.^27^^,^^28^ Age-related microvascular dysfunction in either tissue can reduce oxygen and nutrient delivery, trigger oxidative stress, and upregulate profibrotic mediators such as VEGF and transforming growth factor-beta.^29^^,^^30^ In the retina, emerging OCT-angiography studies have shown capillary nonperfusion and enlargement of the foveal avascular zone in eyes with iERM, supporting a role for chronic hypoxia in Müller-cell activation and subsequent epiretinal membrane formation.^31^, ^32^, ^33^ A similar ischemic environment in the cochlea contributes to hair-cell loss and progressive hearing decline.^34^^,^^35^ These parallel susceptibilities strengthen the hypothesis that microvascular perfusion abnormalities represent a common pathogenic thread linking ERM and sensorineural hearing loss.

This study highlights etiologic heterogeneity in ERM and demonstrates substantial overlap in systemic comorbidity profiles between iERM and sERM. While the traditional classification of secondary and idiopathic remains clinically useful for identifying established local ocular risk factors, these results suggest that the presence or absence of a documented local precipitant may not fully capture systemic susceptibility that could influence ERM development across both categories. In sERM, local ocular pathology likely serves as an initiating trigger, whereas systemic cardiometabolic and inflammatory conditions may modulate the propensity for fibrotic remodeling or the persistence of epiretinal membrane formation. In iERM, similar systemic factors may contribute even when a local precipitant is not documented or clinically apparent in the EHR. Furthermore, these results are hypothesis-generating and point to systemic pathways that warrant further mechanistic investigation in ERM pathogenesis.

Our finding that sERM shares many systemic comorbidities with iERM does not imply that local pathology is unimportant; rather, it supports a model in which systemic cardiometabolic and inflammatory conditions may modulate the magnitude or persistence of the fibrotic response once a local ocular trigger is present. In this framework, local retinal pathology initiates injury and repair cascades, while systemic microvascular dysfunction, chronic low-grade inflammation, and oxidative stress may lower the threshold for ERM formation or progression by compromising barrier integrity and leading tissue repair toward fibrosis. This interaction could help explain why some patients develop clinically significant ERM after similar local events whereas others do not.

This study provides novel insights into the systemic associations of iERM using a large-scale, demographically diverse cohort from the All of Us Research Program. The strengths of this big data approach lie in its robust sample size and access to richly phenotype EHRs. However, our analysis was limited to the binary presence or absence of ERM, without accounting for disease severity or its correlation with visual function. As such, we were unable to assess whether the identified comorbidity clusters are associated with clinically significant, vision-threatening ERM. Importantly, the classification of iERM in this study reflects the absence of documented secondary ocular diagnoses within the EHR and does not exclude the possibility of unrecorded or subclinical ocular pathology. Electronic health record observation time and health care utilization were not adjusted for, which may affect diagnosis capture and partially explain differences in recorded comorbidity burden.

Moving forward, longitudinal studies are needed to assess the causal relationships between these systemic conditions and ERM development, while incorporating minimum observation windows, utilization measures, and comorbidity burden indices to better distinguish biological associations from documentation patterns. Future prospective studies should also evaluate whether systemic risk modification or targeted interrogation of inflammatory pathways is associated with ERM incidence, progression, or postoperative outcomes. While the associations identified here provide evidence for shared pathophysiological mechanisms, prospective studies will help determine whether these comorbidities directly contribute to ERM formation or simply co-occur due to shared risk factors.
