# Supplementary material for: Novel Systemic Associations of Idiopathic Epiretinal Membrane Identified via Machine Learning
Source: Ophthalmol Sci. 2026 Feb 18;6(5):101124. doi: 10.1016/j.xops.2026.101124 (PMC13019322; doi:10.1016/j.xops.2026.101124)
Supplement: Table S1 [file mmc1.pdf]

Supplementary Table 1. Occlusion Factors between Secondary non idiopathic ERM and Idiopathic ERM

**Listed Secondary Causes of ERM**
Acute anterior uveitis
Acute endophthalmitis
Acute hydrops keratoconus
Acute posterior multifocal placoid pigment epitheliopathy
Acute zonal occult outer retinopathy
Transient occlusion of left retinal artery
Transient occlusion of right retinal artery
Corneal abscess
Acquired pit of optic disc
Adhesion of ciliary body
Angle recession
Secondary angle-closure glaucoma
Aphakia
Chorioretinal scar
Choroidal detachment
Choroidal fold
Choroidal infarct
Choroidal retinal neovascularization
Complete luxation of lens
Coloboma of eye
Congenital anomaly of posterior segment of eye
Oculocutaneous albinism
Ocular albinism
Primary cyst of pars plana
Acquired anterior capsular pigmentation
Anterior capsule opacification following extraction of cataract
Secondary cataract

Cataract due to inflammatory disorder
Cataract of left eye due to and following trauma
Cataract of right eye due to and following trauma
Cataract with neovascularization
Localized traumatic opacity
Traumatic cataract
Choroidal degeneration
Degeneration of retina
Pigment dispersion syndrome
Pseudoexfoliation of right lens capsule
Pseudoexfoliation of left lens capsule
Siderosis of eye
Displacement of intraocular lens
Exudative retinopathy
Foreign body in anterior segment of eyeball
Hamartoma of retina
Hemangioma of retina
Endophthalmitis
Hypopyon
Ocular sarcoidosis
Ocular syphilis
Ocular toxoplasmosis
Retinitis
Scleritis
Tuberculosis of eye
Uveitis
Vitritis
Injury of globe of eye
Intraocular hemorrhage
Chorioretinal tumor

Neoplasm of uveal tract
Partial retinal artery occlusion
Partial thickness macular hole of left eye
Proliferative retinopathy
Recession of chamber angle
Retained foreign body in eye
Retinal arteriovenous dilatation
Retinal cyst
Retinal defect
Retinal detachment
Retinal edema
Retinal neovascularization
Retinal embolus
Retinal pigment epithelial abnormality
Retinal vascular occlusion
Retinal telangiectasia
Retinal venous engorgement
Rupture of globe
Scleral staphyloma
Subluxation of lens
Thrombosis of retinal vein
Transient arterial retinal occlusion
Vitreo-retinal adhesion
Anterior scleritis
Retinopathy due to diabetes mellitus
Retinopathy due to type 2 diabetes mellitus
Retinopathy due to type 1 diabetes mellitus
Vitreous hemorrhage due to diabetes mellitus
Choroidal and/or chorioretinal disorder
Disorder of vitreous body and/or retina

Foreign body in posterior wall eye
Posterior scleritis
Disorder of uveal tract
Infectious endophthalmitis
Fungal infection of eye
Infectious secondary iritis
Infective scleritis
Viral retinitis
Herpes zoster scleritis
Herpetic iridocyclitis
Glaucoma with intraocular hemorrhage
Glaucoma associated with ocular trauma
Secondary glaucoma
Hypotony of eye
Ocular ischemic syndrome
Ocular late syphilis
Retina and choroid finding
Acute anterior uveitis
Acute endophthalmitis
Acute hydrops keratoconus
Acute posterior multifocal placoid pigment epitheliopathy
Acute zonal occult outer retinopathy
Transient occlusion of left retinal artery
Transient occlusion of right retinal artery
Corneal abscess
Acquired pit of optic disc
Adhesion of ciliary body
Angle recession
Secondary angle-closure glaucoma
Aphakia

Chorioretinal scar
Choroidal detachment
Choroidal fold
Choroidal infarct
Choroidal retinal neovascularization
Complete luxation of lens
Coloboma of eye
Congenital anomaly of posterior segment of eye
Oculocutaneous albinism
Ocular albinism
Primary cyst of pars plana
Acquired anterior capsular pigmentation
Anterior capsule opacification following extraction of cataract
Secondary cataract
Cataract due to inflammatory disorder
Cataract of left eye due to and following trauma
Cataract of right eye due to and following trauma
Cataract with neovascularization
Localized traumatic opacity
Traumatic cataract
Choroidal degeneration
Degeneration of retina
Pigment dispersion syndrome
Pseudoexfoliation of right lens capsule
Pseudoexfoliation of left lens capsule
Siderosis of eye
Displacement of intraocular lens
Exudative retinopathy
Foreign body in anterior segment of eyeball
Hamartoma of retina

Hemangioma of retina
Endophthalmitis
Hypopyon
Ocular sarcoidosis
Ocular syphilis
Ocular toxoplasmosis
Retinitis
Scleritis
Tuberculosis of eye
Uveitis
Vitritis
Injury of globe of eye
Intraocular hemorrhage
Chorioretinal tumor
Neoplasm of uveal tract
Partial retinal artery occlusion
Partial thickness macular hole of left eye
Proliferative retinopathy
Recession of chamber angle
Retained foreign body in eye
Retinal arteriovenous dilatation
Retinal cyst
Retinal defect
Retinal detachment
Retinal edema
Retinal neovascularization
Retinal embolus
Retinal pigment epithelial abnormality
Retinal vascular occlusion
Retinal telangiectasia

Retinal venous engorgement
Rupture of globe
Scleral staphyloma
Subluxation of lens
Thrombosis of retinal vein
Transient arterial retinal occlusion
Vitreo-retinal adhesion
Anterior scleritis
Retinopathy due to diabetes mellitus
Retinopathy due to type 2 diabetes mellitus
Retinopathy due to type 1 diabetes mellitus
Vitreous hemorrhage due to diabetes mellitus
Choroidal and/or chorioretinal disorder
Disorder of vitreous body and/or retina
Foreign body in posterior wall eye
Posterior scleritis
Disorder of uveal tract
Infectious endophthalmitis
Fungal infection of eye
Infectious secondary iritis
Infective scleritis
Viral retinitis
Herpes zoster scleritis
Herpetic iridocyclitis
Glaucoma with intraocular hemorrhage
Glaucoma associated with ocular trauma
Secondary glaucoma
Hypotony of Eye
Ocular Ischemic Syndrome

Ocular Late Syphillis
Retina and Choroid Finding
