# Supplementary material for: Novel Systemic Associations of Idiopathic Epiretinal Membrane Identified via Machine Learning
Source: Ophthalmol Sci. 2026 Feb 18;6(5):101124. doi: 10.1016/j.xops.2026.101124 (PMC13019322; doi:10.1016/j.xops.2026.101124)
Supplement: Table S2 [file mmc2.pdf]

Supplementary Table 2. Silhouette and inertia of different k clusters

**k-cluster count**	**Silhouette**	**Inertia**
2	0.257	19634
3	0.142	18988
4	0.104	18566
5	0.061	18185
6	0.049	17884
7	0.038	17643
8	0.37	17341
